# Soft drink and non-caloric soft drink intake and their association with blood pressure: the Health Workers Cohort Study

**DOI:** 10.1186/s12937-022-00792-y

**Published:** 2022-06-07

**Authors:** Rubí Hernández-López, Francisco Canto-Osorio, Dèsirée Vidaña-Pérez, Leticia Torres-Ibarra, Berenice Rivera-Paredez, Katia Gallegos-Carrillo, Rafael Velazquez, Paula Ramírez, Tonatiuh Barrientos-Gutiérrez, Jorge Salmerón, Nancy López-Olmedo

**Affiliations:** 1 Office of Health Plan Analysis, Health Plan Technical Deputy Management, Health Plan Administration Management, Directorate General of Administration, Bank of Mexico, Mexico City, Mexico; 2 Center for Population Health Research, National Institute of Public Health, Cuernavaca, Morelos Mexico; 3 Research Center On Policies, Population and Health, Faculty of Medicine, National Autonomous University of Mexico, Mexico City, Mexico; 4 Epidemiological Research and Health Services Unit, Mexican Institute of Social Security, Cuernavaca, Mexico; 5 National Institute of Genomic Medicine, Mexico City, Mexico

**Keywords:** Blood pressure, Soft drink, Non-caloric soft drink, Prospective cohort, Mexico

## Abstract

**Background:**

A few prospective studies have investigated the potential association of soft drink and non-caloric soft drink intake with high blood pressure using methods that adequately consider changes in intake over time and hypertensive status at baseline.

**Objective:**

To prospectively examine the association of soft drink and non-caloric soft drink intake with systolic and diastolic blood pressure in a sample of Mexican adults, overall and by hypertension status.

**Methods:**

We used data from the Health Workers Cohort Study spanning from 2004 to 2018 (*n* = 1,324 adults). Soft drink and non-caloric soft drink intake were assessed with a semiquantitative food frequency questionnaire. We fit multivariable-adjusted fixed-effects models to test the association of soft drink and non-caloric soft drink intake with systolic and diastolic blood pressure. The models were adjusted for potential confounders and considering the potential modifying effect of hypertension status at baseline.

**Results:**

A one-serving increase in soft drink intake was associated with a 2.08 mm Hg (95% CI: 0.21, 3.94) increase in systolic blood pressure and 2.09 mm Hg (95% CI: 0.81, 3.36) increase in diastolic blood pressure over ten years. A stronger association between soft drink intake and diastolic pressure was observed among participants with versus without hypertension at baseline. We found no association between non-caloric soft drink intake and blood pressure.

**Conclusions:**

Our findings support the hypothesis that soft drink intake increases blood pressure. While further studies should be conducted to confirm our findings, food policies and recommendations to limit soft drink intake are likely to help reduce blood pressure at the population level. We probably did not find an association between non-caloric soft drink intake and blood pressure because of the low consumption of this type of beverage in the cohort. More studies will be needed to understand the potential effect of non-caloric beverages on blood pressure.

**Supplementary Information:**

The online version contains supplementary material available at 10.1186/s12937-022-00792-y.

## Introduction

Hypertension is one of the main causes of global morbidity and mortality. In 2019, the number of adults (30–79 years) with hypertension reached 652 million for men and 626 million for women [1]. Hypertension has been associated with stroke, renal failure, and death and is an important cause of disability-adjusted life years [2, 3]. It is therefore relevant to identify the factors that can reduce the risk of hypertension.

The intake of sugar-sweetened beverages (SSBs) has been associated with weight gain [4, 5], type-2 diabetes [6], and coronary heart disease [6–8]. Moreover, animal studies found that high SSBs intake can induce hypertension [9, 10], and prospective studies support the hypothesis of a positive association between SSBs intake and high blood pressure and hypertension [11–16]. Two main mechanisms have been proposed to explain the association between soft drink intake and blood pressure. First, the potential role of high-fructose corn syrup since a high intake of fructose has been associated with increases in blood pressure, adipogenesis, and oxidative stress. Also, fructose favors fat accumulation and induces vascular damage [17, 18], and animal studies indicate that high fructose intake seems to alter the hemodynamic system [19]. The second mechanism is the potential increase in blood pressure by soft drinks through weight gain due to the low to null caloric compensation induced by liquid calories, low satiety, and high added sugar content [20, 21].

Unlike regular soft drinks, the mechanisms for which non-caloric soft drink intake may change blood pressure are not clear. Animal studies suggest that aspartame, a non-caloric sweetener usually found in non-caloric soft drinks, has antihypertensive properties due to its metabolite, tyrosine [22]. Also, in animal studies, sucralose and acesulfame potassium showed to cause renal damage and endothelial dysfunction, which may result in HTN and increased blood pressure variability [23]. However, these effects have not been observed in humans. Also, non-caloric soft drinks have been proposed as an alternative to avoid an increase in blood pressure through weight gain [24]. However, weight gain could also occur with the consumption of non-caloric soft drinks. As with other liquids, non-caloric soft drinks might have less impact on satiety than do solid foods [25]. Also, non-caloric soft drinks’ sweetness may increase appetite [25]. It has also been proposed that the intake of non-caloric soft drinks may result in energy compensation or overcompensation [26].

Cohort studies have analyzed the association of soft drink and non-caloric soft drink intake with hypertension by linking soft drink intake at baseline to changes in blood pressure or hypertension over time. It has been estimated that each additional serving of SSBs per day increases hypertension risk by 8% [12]. Still, studies are subject to several confounding factors and do not consider changes in intake over time [27]. The use of econometric fixed-effects models can improve the association estimates by removing time-invariant confounders and providing estimates of how much blood pressure changes as soft drink intake does [27]. Also, the effects of soft drinks could be stronger among people with hypertension, yet, no study has formally tested the possibility of diagnosed hypertension being an effect modifier in the association between blood pressure and soft drink intake. Finally, it has been proposed that type-2 diabetes and obesity could mediate the association between soft drink intake and blood pressure. Yet, only one study in adolescents has analyzed the possibility of BMI (body mass index) as a mediator [28].

Mexico is the second-largest consumer of soft drinks in the world [29]. Also, 25.5% of Mexican adults have hypertension, with only 45.6% under control [30]. This context provides sufficient variability to explore the potential impact of soft drink intake on blood pressure. We aimed to prospectively examine the association between changes in soft drink and non-caloric soft drink intake and changes in systolic and diastolic blood pressure among Mexican adults, using fixed-effects models. We hypothesized that increases in soft drink and non-caloric soft drink intake would be associated with increases in systolic and diastolic blood pressure. Additionally, we evaluated the potential modifier effect of hypertension in the association of soft drink and non-caloric soft drink intake with blood pressure. Finally, we explored the association of this type of beverage with blood pressure in participants without type-2 diabetes or obesity at baseline.

## Methods

### Study design and participants

The Health Workers Cohort Study (HWCS) is an ongoing open cohort study that recruited employees from the Social Security Institute in Mexico (IMSS for its Spanish acronym) and their relatives. Details of the study design have been reported elsewhere [31]. For the present study, we used data collected during the first (2004–2006), second (2010–2012), and third waves (2016–2018).

**Fig. 1:**
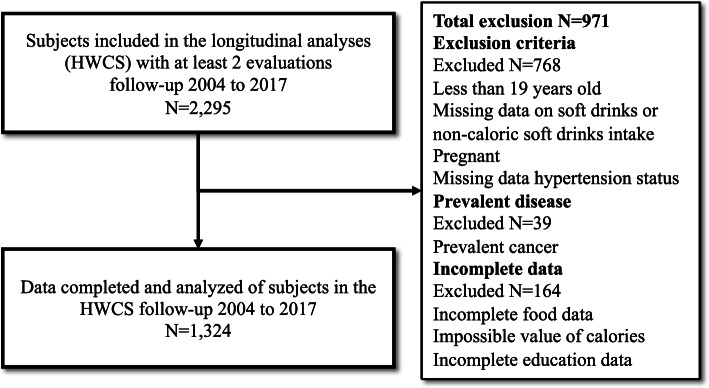
Consort diagram

We included respondents that had participated in at least two waves (*n* = 2,295). We excluded participants under 19 years of age, pregnant, or with missing data on soft drink intake or blood pressure (*n* = 768). Adults with cancer were also excluded (*n* = 39), given its potential effect on appetite and diet. Finally, we excluded participants with incomplete education data, with answers in less than 75% of the semi-quantitative food frequency questionnaires (SFFQ), with an entire section of the food frequency questionnaire left in blank, or with total energy intakes < 500 kcal/day or > 6500 kcal/day (*n* = 164) [32] (Fig. 1).

### Study measurements

#### Blood pressure

Trained personnel collected systolic and diastolic blood pressure measurements following standard procedures and techniques [33] using an automatic digital monitor (OMROM HEM-907).

#### Soft drinks

We assessed soft drink and non-caloric soft drink intake using the 116-item SFFQ, previously validated in the Mexican population [34]. For each food, participants reported the frequency and the number of standard portions they consumed over 12 months prior to the interview. The possible responses of frequency were never, < 1 time/month, 1–3/month, 1, 2–4, 5–6 times/week, or 1, 2–3, 4–5, 6 or more times/daily. We defined soft drinks as cola and fruit-flavored non-cola carbonated beverages (hereinafter cola and flavored soft drinks). Non-caloric soft drinks were defined as cola soft drinks and flavored carbonated soft drinks with non-caloric sweeteners. We established the standard serving in 355 ml for both soft drinks and non-caloric soft drinks.

#### Covariates

The HWCS captured diverse information through questionnaires; for our analyses, we considered demographic characteristics (age, sex, and educational level), lifestyle habits (total energy intake, diet, alcohol intake, smoking status, and physical activity), and medical history (body mass index, type-2 diabetes and hypertension status). We categorized educational level as basic school, high school, undergraduate, and graduate or more, and smoking status as current, former, or never smoker. We estimated alcohol intake (g/day and tertiles) and total energy intake (kcal/day) from the SFFQ. We computed the DASH score based on the traditional system developed by Fung et al., as previously described in detail [35]. Briefly, the scoring system is based on quintile categories. For recommended components (whole grains, fruits, vegetables, low-fat dairy products, and nuts and legumes), those in the lowest quintile of intake receive 1 point, and those in the highest quintile receive 5 points. In contrast, for components for which lower intakes are recommended (red and processed meats, sweetened beverages, and sodium), those in the highest and lowest quintile of intake receive 1 and 5 points, respectively. We then summed up the component scores to obtain an overall DASH score ranging from 8 to 40. Physical activity during leisure time was assessed by using a validated physical activity questionnaire [36]. We classified participants as active based on WHO recommendation (physical activity > 150 min per week) [37]. Type-2 diabetes and hypertension status were considered dichotomous variables (yes/no). Participants were classified with type-2 diabetes or hypertension if they reported having been diagnosed with any of these diseases or used medications to treat them.

We also considered weight and height measurements. Trained nurses carried out these measurements following standard procedures and techniques [38]. Weight was measured with a calibrated electronic scale (Tanita, model BC-533) and height with stadiometers (Seca). The BMI was calculated using the standard equation [BMI = weight (kg)/height (m^2^)] and categorized based on WHO definition [39].

### Statistical analysis

We conducted a descriptive analysis of baseline characteristics by soft drink and non-caloric soft drink intake categories (never, < 1 time/week, 1–4 times/week, and > 5 times/week) using means and standard deviations, medians and interquartile ranges, or percentages. To estimate the rates of change over time, we calculated the change for blood pressure, dietary variables, physical activity, and BMI for every ten years in the cohort, using unadjusted linear regression models. Fixed-effects models were used to analyze the association of soft drink intake with systolic and diastolic blood pressure. We first adjusted models for time elapsed since baseline assessment and rescaled them to represent 10-year intervals considering that blood pressure changes occur gradually. We added interactions of the time since baseline with sex, age and BMI (centered at their baseline mean) to allow for sex, age and BMI-at-baseline slopes. Models were then further adjusted for total energy intake, tertiles of alcohol intake, physical activity, smoking status, and educational level. Marginal blood pressure means in adults consuming 0-, 1- and 2-units of soft drinks and non-caloric soft drinks per day at baseline and 10 years after participating in the cohort were calculated using the results from the fixed-effects model. Finally, we assessed whether the association of soft drink and non-caloric soft drink intake with systolic and diastolic blood pressure was different by self-reported hypertension status at baseline. For this purpose, we fitted models with a triple interaction that included interaction terms between time, soft drink or non-caloric soft drink intake, and hypertension status. The equations of the models are available in the supplementary material.

### Sensitivity analysis

To test the potential differential effect of type-2 diabetes on the association of soft drink and non-caloric soft drink intake with blood pressure, we selected all participants who had no diagnosis of type-2 diabetes at baseline. We fitted a full model that included the variable of type-2 diabetes diagnosis (yes/no) during follow-up (*n* = 1,241). We conducted a similar procedure for obesity (*n* = 1,071). We performed all the analyses using Stata 14.0 (StataCorp, Stata Statistical Software, Release 14, 2015).

## Results

The sample consisted of 1,324 participants, which were followed for a maximum of 13.7 y, with an average of 8.7 y. Mainly non-smoking women, more than half (51.7%) consumed 1–4 servings of soft drinks per week and 21.0% more than 5 servings per week. About 70% reported not being consumers of non-caloric soft drinks. The participants in the highest level of soft drink and non-caloric soft drink intake had higher total energy intakes than individuals with lower intakes. Also, we observed a smaller percentage of physically active participants among higher consumers of soft drinks and non-caloric soft drinks. The prevalence of obesity was higher as soft drink intake increased. Similarly, the prevalence of obesity was higher in participants that reported consuming non-caloric soft drinks in some frequency than those that never have consumed (Table 1).

**Table 1:** Characteristics of participants in the Health Workers Cohort Study according to their soft drink and non-caloric soft drink intake at baseline (*n* = 1,324)

		**Soft drink intake**			**Non-caloric soft drink intake**			
		*n* = 1324						
	Never	< 1 per week	1–4 per week	> 5 per week	Never	< 1 per week	1–4 per week	> 5 per week
	*N* = 77(5.82)	*n* = 285(21.5)	*n* = 684(51.7)	*n* = 278(21.0)	*n* = 933(70.5)	*n* = 252(19.0)	*n* = 95(7.2)	*n* = 44(3.3)
Sex, %								
Women	90.91	89.12	81.73	67.27	80.6	77.8	87.4	88.6
Age (years)^a^	50.7(12.1)	47.4(13.3)	45.3(12.7)	43.5(11.8)	45.7(13.0)	45.1(12.7)	47.1(10.7)	44.7(11.7)
Systolic Blood pressure (mm Hg)^a^	116.9(13.8)	115.2(12.6)	116.1(13.0)	117.5(14.0)	116.0(13.3)	117.7(13.3)	115.5(12.4)	113.8(11.7)
Diastolic Blood pressure (mm Hg)^a^	71.9(11.6)	70.7(9.3)	71.7(9.2)	73.1(12.0)	71.5(10.0)	72.5(10.0)	72.9(9.9)	72.8(10.0)
Total energy intake (kcal/day)^a^	2146.5(938.1)	1998.2(876.8)	2085.1(860.9)	2407.3(869.5)	2148.6(909.0)	2124.2(838.8)	2006.5(786.8)	2292.2(690.7)
Alcohol (g/day)^b^	0.2(0.0–0.8)	0.6(0–1.7)	1.0(0.2–4.0)	1.5(0.4–5.6)	0.8(0.4–2.6)	1.6(0.3–5.6)	1.8(0.6–6.8)	1.6(0.3–3.0)
Tertile 1 (< 0.6), %	57.1	44.9	31.7	26.3	38.9	26.2	22.1	27.3
Tertile 2 (0.6–2.4), %	27.3	33.7	33.3	35.6	33.9	32.1	33.7	34.1
Tertile 3 (> 2.4), %	15.6	21.4	34.9	38.1	27.2	41.7	44.2	38.6
Physical activity (hrs. per week)^b^	1.5(0.4–5.3)	1.5(0.4–3.9)	1.5(0.4–3.7)	0.8(0.2–3.6)	1.5(0.3–3.8)	1.5(0.4–4.3)	1.5(0.2–4.0)	1.5(0.4–5.6)
Active (≥ 2.5 h. per week), %	42.9	42.5	37.4	31.7	37.8	37.7	37.9	31.8
Smoking								
Never,%	64.9	66.3	59.1	48.9	62.6	53.2	44.2	43.2
Former,%	6.5	10.2	16.1	22.3	13.6	17.9	22.1	31.8
Current,%	28.6	23.5	24.9	28.4	23.8	29.0	33.7	25.0
Education								
Basic school, %	11.7	16.1	10.7	10.1	12.5	9.5	11.6	9.1
High school, %	13.0	14.4	16.4	18.0	17.8	12.7	10.5	11.4
Undergraduate, %	10.4	17.2	27.2	25.9	22.9	23.8	32.6	22.7
Graduate or more, %	64.9	52.3	45.8	46.0	46.7	54.0	45.3	56.8
BMI(kg/m^2^)^a^	26.0(4.0)	26.0(4.4)	26.7(4.4)	26.7(4.8)	26.2(4.5)	27.2(4.3)	27.9(4.0)	27.0(4.7)
Normal,%	44.2	48.1	37.9	39.2	44.5	32.1	26.3	40.9
Overweight,%	39.0	36.1	42.4	39.2	37.9	46.4	46.3	38.6
Obesity,%	16.9	15.8	19.7	21.6	17.6	21.4	27.4	20.5
Type 2 diabetes, %	18.2	13.0	6.6	3.2	6.9	9.9	11.6	11.4

^a^Mean (SD)

^b^p50 (p25-p75)

When we analyze the changes in continuous variables for every 10 years in the cohort, we found that the mean systolic and diastolic blood pressure increased 5.65 and 7.59 mmHg, respectively, as did BMI (1.2 kg/m^2^). Reductions were observed in soft drink intake (-0.12 serving/day), non-caloric soft drink intake (-0.01 serving/day) and total energy intake (-426 kcal/day) (Table 2).

**Table 2:** Changes in continuous variables for every 10 years in the cohort (*n* = 1,324)

	**Baseline**	**Change**	***P*-value**
Systolic Blood pressure (mmHg)^a^	116.2(13.2)	5.65	< 0.001
Diastolic Blood pressure (mmHg)^a^	71.8(10.0)	7.59	< 0.001
Soft drink intake (servings/day)^a^	0.4(0.7)	-0.12	0.028
Non-caloric soft drink intake (servings/day)^a^	0.1(0.4)	-0.01	0.904
Total energy intake (kcal/day)^a^	2128.6(877.7)	-425.7	< 0.001
Alcohol (g/day)^b^	0.8(0.2–3.2)	-0.6	0.421
Physical activity (hrs. per week)^b^	1.5(0.4–3.9)	-0.5	0.132
BMI (kg/m^2^)^a^	26.5(4.5)	1.2	< 0.001

^a^Mean (SD)

^b^p50 (IQR)

**Fig. 2:**
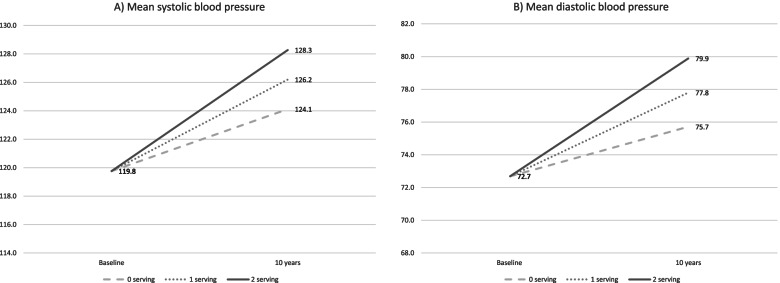
Mean systolic blood pressure (**A**) and diastolic blood pressure (**B**) at baseline and after 10 years. Legend: Mean systolic blood pressure (**A**) and diastolic blood pressure (**B**) at baseline and after 10 years. in adults consuming 0 servings of soft drinks at baseline and then maintained their intake or increased it by 1 or 2 servings. Fixed-effects models were used to predict mean diastolic blood pressure adjusting for baseline age centered to mean, sex centered, body mass index centered to mean, physical activity, smoking status, alcohol intake, education, and energy intake

Soft drink intake was positively associated with systolic blood pressure in models adjusted for age and sex and in fully adjusted models. In the full model, a one-serving increase in soft drink intake was associated with 2.08 mm Hg increase in systolic blood pressure (95% CI: 0.21, 3.94) during 10 years (Table 3). To facilitate the interpretation of these coefficients, we present the expected mean blood pressure for participants 10 years after baseline, assuming all drank 0 servings of soft drink at baseline and then maintained their intake or increased it by 1 or 2 servings (Fig. 2). After ten years, those who maintained a zero intake of soft drinks would have had a systolic blood pressure of 124.1 mmHg, compared to 126.2 mmHg and 128.3 mmHg among those who increased their intake to 1 and 2 servings of soft drinks, respectively. The intake of non-caloric soft drinks was not associated with systolic blood pressure (Table 3).

**Table 3:** Association of soft drink and non-caloric soft drink intake with systolic and diastolic pressure (*n* = 1,324)

	**Soft drink intake**						**Non-caloric soft drink intake**					
	**Model 1**^a^			**Model 2**^b^			**Model 1**^a^			**Model 2**^b^		
**Variable**	**Coefficient**	**CI 95%**	***P*-value**	**Coefficient**	**CI 95%**	***P*-value**	**Coefficient**	**CI 95%**	***P*-value**	**Coefficient**	**CI 95%**	***P*-value**
**Systolic blood pressure**												
Soft drinks	0.39	-0.98, 1.77	0.57	0.18	-1.23, 1.60	0.80	-0.82	-3.40,1.73	0.52	-0.93	-3.50, 1.64	0.48
Time	4.25	2.38, 6.11	< 0.001	10.36	6.73, 13.98	< 0.001	4.89	3.24, 6.55	< 0.001	11.46	7.96, 14.95	< 0.001
Soft drinks × Time	2.26	0.44, 4.09	0.02	2.08	0.21, 3.94	0.03	0.73	-1.42, 2.89	0.507	0.83	-1.34, 2.99	0.46
**Diastolic blood pressure**												
Soft drinks	0.42	-0.52, 1.39	0.39	0.23	-0.74, 1.20	0.64	-0.05	-1.81, 1.71	0.95	-0.07	-1.85, 1.70	0.94
Time	5.19	3.91, 6.46	< 0.001	4.80	2.31, 7.28	< 0.001	5.83	4.70, 6.97	< 0.001	5.68	3.25, 8.10	< 0.001
Soft drinks × Time	2.02	0.77, 3.26	0.01	2.09	0.81, 3.36	0.001	0.08	-1.39, 1.57	0.90	0.15	-1.35, 1.65	0.84

^a^Model 1: Soft drinks and BP, adjusted for baseline age centered to mean, sex centered

^b^Model 2: Model 1 plus body mass index centered to mean, physical activity, smoking status, alcohol intake, education, energy intake and

Soft drink intake was also positively associated with diastolic blood pressure. In the full model, a one-serving increase in soft drink intake was associated with 2.09 mm Hg (95% CI: 0.81, 3.36) increase in diastolic blood pressure (Table 3). After 10 years, those who maintained a zero soft drink intake would have had 75.7 mm Hg of diastolic blood pressure, compared to 77.8 mm Hg and 79.9 mm Hg among those who increased their intake to 1 or 2 servings of soft drinks (Fig. 2). As observed for systolic blood pressure, non-caloric soft drink intake was not associated with diastolic blood pressure.

The association between soft drink intake and systolic blood pressure was not different by hypertension status. The diastolic blood pressure was 4.24 mm Hg (95% CI: 1.28, 7.20) higher in participants with versus without hypertension for each 1-unit increase in soft drink intake over 10 years (Table 4). The estimated association of soft drinks with systolic and diastolic blood pressure in participants without type-2 diabetes or obesity at baseline was similar when the models were further adjusted for these diseases (Supplementary tables 1–2). We did not find that hypertension, type-2 diabetes, or obesity status differentiated the association between non-caloric soft drink intake and blood pressure (Table 4 and Supplementary tables 1–2).

**Table 4:** Association of soft drink and non-caloric soft drink intake with systolic and diastolic blood pressure by hypertension status at baseline (*n* = 1,324)^a^

	**Soft drink intake**			**Non-caloric soft drink intake**		
**Variable**	**Coefficient**	**CI 95%**	***P*-value**	**Coefficient**	**CI 95%**	***P*-value**
**Systolic blood pressure**						
Soft drinks	0.22	-1.34, 1.79	0.78	-0.35	-3.16, 2.47	0.80
Time	10.26	6.49, 14.03	< 0.001	10.83	7.20, 14.45	< 0.001
Soft drinks × Time	1.50	-0.66, 3.66	0.17	1.55	-1.42, 4.54	0.31
HTA status	-2.40	-5.10, 0.29	0.08	-2.30	-4.79, 0.18	0.07
Soft drinks × HTA status	0.13	-2.95, 3.21	0.93	-3.95	-10.14, 2.23	0.21
HTA status × Time	0.80	-2.24, 3.85	0.60	1.63	-1.07, 4.33	0.24
Soft drinks × Time × HTA status	2.09	-2.24, 6.42	0.34	1.67	-3.68, 7.01	0.54
**Diastolic blood pressure**						
Soft drinks	0.59	-0.48, 1.66	0.28	-0.16	-2.11, 1.79	0.87
Time	5.71	3.13, 8.29	< 0.001	5.83	3.32, 8.33	< 0.001
Soft drinks × Time	1.07	-0.41, 2.54	0.16	0.99	-1.08, 3.05	0.35
HTA status	-0.58	-2.43, 1.26	0.53	-1.22	-2.95, 0.49	0.16
Soft drinks × HTA status	-1.50	-3.60, 0.60	0.16	-0.38	-4.66, 3.90	0.86
HTA status × Time	-2.22	-4.30, -0.14	0.04	-0.27	-2.15, 1.60	0.77
Soft drinks × Time × HTA status	4.24	1.28, 7.20	< 0.01	-0.60	-4.30, 3.10	0.75

^a^Models adjusted for age centered to mean, sex centered, body mass index centered to mean, physical activity, smoking status, alcohol intake, education and energy intake

## Discussion

We aimed to estimate the effect of changes in soft drink and non-caloric soft drink intake on changes in systolic and diastolic blood pressure. We analyzed a cohort of health professionals with a median of 9.19 years of follow-up. We found a positive association between the number of servings of soft drinks and systolic and diastolic blood pressure (2.08 mm Hg and 2.09 mm Hg increase in systolic and diastolic blood pressure, respectively), by increasing one serving of soft drink per day during 10 years in the cohort. We also found that the estimated associations differed by hypertensive status at baseline for diastolic but not systolic blood pressure. Finally, the estimations remained similar after adjusting for type-2 diabetes status and obesity in participants without these diseases at baseline.

To our knowledge, only one study has assessed how SSBs intake is associated with blood pressure over time. In adults who participated in an 18-month intervention trial, Chen et al., found a reduction of 1.8 mm Hg of systolic blood pressure and 1.1 mm Hg of diastolic blood pressure by reducing 1 serving (355 mL) of SSBs per day [15]. There are several potential reasons we found an association between soft drink intake and blood pressure of a smaller magnitude if we compare the same period. First, Chen, et al., applied mixed-effects models to estimate blood pressure changes in responding to changes in SSBs. This method, in contrast to fixed-effects models, uses both within- and between-individual exposure-outcome associations, increasing the potential variance to be explained (i.e., the effect of soft drinks on blood pressure within each individual as in fixed-effects, plus the differences in blood pressure across different types of soft drink consumers) but at the expense of potentially introducing time-invariant confounding [26]. Second, the study mentioned above analyzed SSBs, which include soft drinks and other sweetened non-carbonated drinks. Third, Chen et al. estimated the association between SSBs and blood pressure using data from a behavioral intervention trial in adults aged 25–79 years with elevated blood pressure or hypertension at baseline, who could display larger blood pressure changes than normotensive participants.

Although of smaller than previous studies, our estimated associations of soft drink intake with systolic and diastolic blood pressure are relevant from a public health perspective. Ng, et al., estimated that the mean purchase of SSBs was 214 mL per capita per day (0.6 portions per day) in Mexican households in areas with more than 50,000 inhabitants [40]. However, 23.4% of the study sample had an estimated mean purchase of SSBs of 322 mL per capita per day, almost one serving per day. We estimated a mean systolic and diastolic blood pressure of 122.4 mm Hg and 76.7 mm Hg, respectively, in those consuming one serving of soft drink per day for the previous ten years. The level of systolic blood pressure falls under the classification of elevated [41]. Moreover, the estimated increase of systolic and diastolic blood pressure over ten years would be 6.2 mm Hg and 5.1 mm Hg, respectively, in adults consuming one serving of soft drinks per day and moving a large proportion of the population towards hypertension. The estimated changes in blood pressure are also clinically relevant, considering that mean reductions of 5 mm Hg in systolic and 2 mm Hg in diastolic blood pressure can reduce cardiovascular diseases in a range of magnitude between 6 and 20% [42, 43].

Our study found no association between non-caloric soft drink intake and changes in blood pressure. Few studies have evaluated the association between artificially sweetened beverages; a prospective study with five thousand subjects included in the Multi-Ethnic Study of Atherosclerosis (MESA) did not find that daily intake of non-caloric soft drinks increased the hypertension risk [44]. Nevertheless, a meta-analysis with four studies (including the MESA study) estimates a relative risk of 1.14 (95% CI 1.10, 1.18) between individuals with the highest versus the lowest consumption of artificially sweetened beverages [16]. We probably did not find an association between non-caloric soft drink intake and blood pressure because of low variability in the exposure, considering that 70% of the sample reported they had never consumed this type of beverage. Moreover, the inclusion of other non-caloric beverages might change the association of these beverages with blood pressure. In any case, more studies will be needed to understand better the potential effect of non-caloric beverages on blood pressure.

Our study has certain limitations that have to be acknowledged. First, given the observational nature of our study, we cannot rule out residual confounding, especially for time-variant variables, such as changes in types of hypertensive medications used. Second, we used an SFFQ to estimate soft drink intake. Some potential sources for error for intake data collected by SFFQ are the portion size estimation and the frequency report. The SFFQ may not capture as much detail about the type of food and beverages compared to other dietary methods. However, we would expect that the potential misclassifications were not differential. Third, our study focused on soft drinks instead of sugar-sweetened beverages. The SFFQ includes other sweetened beverages (e.g., flavored waters and juices) but not for all the waves since they were added to the instrument over time. Moreover, the SFFQ does not include separate items to differentiate industrialized from homemade beverages. Therefore, it is unknown if natural or artificial sweetener was added to homemade beverages. Given the uncertainty of knowing the nutrient composition of these beverages, we decided to focus our study on carbonated drinks. Moreover, soft drinks are the sugar-sweetened beverages more consumed in Mexico [45]. Fourth, changes in soft drinks intake might be explained by modifications in the SFFQ. However, it is unlikely this can explain, at least partially, the findings of our study since the way to ask for these beverages did not change in the three waves. Therefore, we assume that the changes in carbonated drinks observed in this study are due to changes in consumption. Fifth, the FFQ used in our study was only validated in Mexican women. Therefore, the instrument may not be adequate to estimate the dietary intake among men, which represent around 20% of the study sample. Six, the wide confidence intervals observe when we analyzed hypertension status at baseline as a potential modifier suggests that the sample was insufficient to conduct models with triple interactions. Future cohort studies with larger samples will be needed to evaluate whether hypertension status modifies the association between soft drink intake and blood pressure. Last, the distribution of Social Security Institute in Mexico workers for 2014 (data not available for previous years) was 60% women and 40% men [46]. This distribution remained constant until 2020 [47]. If we assume that the same distribution was for 2004 (the first wave of our study), perhaps our sample does not represent the distribution from which it was obtained. The latter may be because either more female health workers or female relatives participated in this study. However, representativeness is not a concern in our study. The overall goal is to add to the evidence about the association between soft drinks and blood pressure by using econometric models that remove time-invariant confounders, including sex.

## Conclusions

In conclusion, we found that a greater intake of soft drinks was associated with higher systolic and diastolic blood pressure independently of type-2 diabetes or excess body weight. Although the changes in blood pressure by soft drink intake seem modest, these could substantially impact cardiovascular disease incidence in the long term. Our results support food policies and recommendations to limit the intake of soft drinks as a public health strategy to prevent and reduce the incidence of hypertension.

## Supplementary Information

**Additional file 1.**

**Additional file 2:** **Supplementary Table 1.** Association of soft drink and non-caloric soft drink intake with blood pressure (mm Hg) in participants without type-2 diabetes at baseline (n=1,241)^1^. **Supplementary Table 2.** Association of soft drink and non-caloric soft drink intake with blood pressure (mm Hg) in participants without obesity at baseline (n=1,071)^1^.

## Data Availability

The datasets used and/or analysed during the current study are available from the corresponding author on reasonable request.

## Abbreviations

BMI: Body mass index
SFFQ: Semi-quantitative food frequency questionnaires
SSB: Sugar sweetened beverages
HWCS: Health workers cohort study
