# Supplementary material for: Soft drink and non-caloric soft drink intake and their association with blood pressure: the Health Workers Cohort Study
Source: Nutr J. 2022 Jun 7;21:37. doi: 10.1186/s12937-022-00792-y (PMC9171938; doi:10.1186/s12937-022-00792-y)
Supplement: Supplementary file 1 — Additional file 1. [file 12937_2022_792_MOESM1_ESM.docx]

**Fixed-effect regression equations**

Note: All the models described were run in the same way for non-caloric soft drink.

***Association of soft drink and non-caloric soft drink intake with systolic and diastolic pressure (n=1,324)***

Model 1.

Y = _0_ + _1_(time_it_) + _2_(soft drink_it_) + _3_(soft drink_it_*time_it_) + _4_(sex centered_it_*time_it_) + _5_(age centered_it_*time_it_) + u_it_

_0_ = Intercept

_1_(Time_it_) = Ten-year trajectory of blood pressure when soft drink intake is 0, adjusting for sex centered, age centered for subject i in the time t

_2_(Soft drink_it_) = Trajectory of blood pressure when time is 0, adjusting for sex centered, age centered for subject i in the time t

_3_(Soft drink_it_*time_it_)= Ten-year trajectory of blood pressure when soft drink intake is 1, for subject i in the time t

_4_(Sex centered_it_*time_it_) = Ten-year trajectory of blood pressure according sex centered for subject i in the time t

_5_(Age centered_it_*time_it_) = Ten-year trajectory of blood pressure according age centered for subject i in the time t

u_it_ = error term

Model 2. Model 1 plus physical activity, smoking status, alcohol intake, education and energy intake.

Y = _0_ + _1_(time_it_) + _2_(soft drink_it_) + _3_(soft drink_it_*time_it_) + _4_(sex centered_it_*time_it_) + _5_(age centered_it_*time_it_) + _6_(bmi centered*time) _7_(education_it_*time_it_) + _8_(calories_it_*time_it_) + 9 n(covariates_it_) + u_it_

_0_ = Intercept

_1_(Time_it_) = Ten-year trajectory of blood pressure when soft drink intake is 0, adjusting for sex centered, age centered, education, calories and covariates for subject i in the time t

_2_(Soft drink_it_) = Trajectory of blood pressure when time is 0, adjusting for sex centered, age centered, education, calories and covariates for subject i in the time t

_3_(Soft drink_it_*time_it_)= Ten-year trajectory of blood pressure intake when soft drink intake is 1, for subject i in the time t

_4_(Sex centered_it_*time_it_) = Ten-year trajectory of blood pressure according sex centered for subject i in the time t

_5_(Age centered_it_*time_it_) = Ten-year trajectory of blood pressure according age centered for subject i in the time t

_6_ (Bmi centered_it_*time_it_) = Ten-year trajectory of blood pressure according bmi centered for subject i in the time t

_7_(Education_it_*time_it_) = Ten-year trajectory of blood pressure according education level for subject i in the time t

_8_(Calories_it_*time_it_) = Ten-year trajectory of blood pressure according calorie intake for subject i in the time t

_n_(Covariates that vary in time) = physical activity, smoking status and alcohol intake.

u_it_ = error term

***Association of soft drink and non-caloric soft drink intake with systolic and diastolic blood pressure by hypertension status at baseline (n=1,324)***

Y = _0_ + _1_(time_it_) + _2_(soft drink_it_) + _3_(hypertension status_it_) + _4_(soft drink_it_*time_it_) + _5_(hypertension status_it_*time_it_) + _6_(soft drink_it_*hypertension status_it_*time_it_) + _7_(sex centered_it_*time_it_) + _8_(age centered_it_*time_it_) + _9_(bmi centered_it_*time_it_) + _10_(education_it_*time_it_) + _11_(calories_it_*time_it_) + n(covariates_it_) + u_it_

_0_ = Intercept

_1_ (Time_it_) = Ten year trajectory of blood pressure when soft drink intake is 0, adjusting for sex centered, age centered, education, calories and covariates for subject i in the time t

_2_ (Soft drink_it_) = Trajectory of blood pressure when time is 0, adjusting for sex centered, age centered, education, calories and covariates for subject i in the time t

_3_(Hypertension status) = Ten-year trajectory of blood pressure when time is 0 and soft drink intake is 0, adjusting for sex centered, age centered, education, calories and covariates for subject i in the time t

_4_(Soft drink_it_*time_it_)= Ten-year trajectory of blood pressure when soft drink intake is 1, for subject i in the time t

_5_(Hypertension status*time) Ten-year trajectory of blood pressure according hypertension status for subject i in the time t

_6_(Soft drink_it_*hypertension status_it_*time_it_) = Ten-year trajectory of blood pressure when soft drink intake is 1 and hypertension status is 1, for subject i in the time t

_7_(Sex centered_it_*time_it_) = Ten-year trajectory of blood pressure according sex centered for subject i in the time t

_8_(Age centered_it_*time_it_) = Ten-year trajectory of blood pressure according age centered for subject i in the time t

_9_(Bmi_it_*time_it_) = Ten-year trajectory of blood pressure according bmi centered for subject i in the time t

_10_(Education_it_*time_it_) = Ten-year trajectory of blood pressure according education level for subject i in the time t

_11_(Calories_it_*time_it_) = Ten-year trajectory of blood pressure according calorie intake for subject i in the time t

_n_(Covariates that vary in time) = physical activity smoking status, alcohol intake.

u_it_ = error term

***Association between soft drinks and non-caloric soft drinks intake and blood pressure (mm Hg) in participants without type-2 diabetes at baseline (n=1,241)***

Y = _0_ + _1_(Time_it_) + _2_(soft drink_it_) + _3_(soft drink_it_*time_it_) + _4_(type 2 diabetes status_it_*time_it_) _5_(sex centered_it_*time_it_) + _6_(age centered_it_*time_it_) + _7_(bmi_it_*time_it_) + _8_(education_it_*time_it_) + _9_(calories_it_*time_it_) + n(covariates_it_) + u_it_

_0_ = Intercept

_1_(Time_it_) = Ten year trajectory of blood pressure when soft drink intake is 0, adjusting for type 2 diabetes status, sex centered, age centered, education, calories and covariates for subject i in the time t

_2_(Soft drink_it_) = Trajectory of blood pressure when time is 0, adjusting for type 2 diabetes status, sex centered, age centered, education, calories and covariates for subject i in the time t

_3_(Soft drink_it_*time_it_)= Ten-year trajectory of blood pressure when soft drink intake is 1, for subject i in the time t

_4_(Type 2 diabetes status*time) Ten-year trajectory of blood pressure according type 2 diabetes status for subject i in the time t

_5_(Sex centered_it_*time_it_) = Ten-year trajectory of blood pressure according sex centered for subject i in the time t

_6_(Age centered_it_*time_it_) = Ten-year trajectory of blood pressure according age centered for subject i in the time t

_7_(Bmi_it_*time_it_) = Ten-year trajectory of blood pressure according bmi centered for subject i in the time t

_8_(Education_it_*time_it_) = Ten-year trajectory of blood pressure according education level for subject i in the time t

_9_(Calories_it_*time_it_) = Ten-year trajectory of blood pressure according calorie intake for subject i in the time t

_n_(Covariates that vary in time) = physical activity, smoking status and alcohol intake.

u_it_ = error term

***Association between soft drinks and non-caloric soft drinks intake and blood pressure (mm Hg) in participants without obesity at baseline (n=1,071)***

Y = _0_ + _1_(Time_it_) + _2_(soft drink_it_) + _3_(soft drink_it_*time_it_) + _4_(body mass index_t_*time_it_) _5_(sex centered_it_*time_it_) + _6_(age centered_it_*time_it_) + _7_(bmi_it_*time_it_) + _8_(education_it_*time_it_) + _9_(calories_it_*time_it_) + n(covariates_it_) + u_it_

_0_ = Intercept

_1_(Time_it_) = Ten year trajectory of blood pressure when soft drink intake is 0, adjusting for body mass index, sex centered, age centered, education, calories and covariates for subject i in the time t

_2_(Soft drink_it_) = Trajectory of blood pressure when time is 0, adjusting for body mass index, sex centered, age centered, education, calories and covariates for subject i in the time t

_3_(Soft drink_it_*time_it_)= Ten year trajectory of blood pressure when soft drink is 1, for subject i in the time t

_4_(Body mass index*time) Ten-year trajectory of blood pressure according body mass index for subject i in the time t

_5_(Sex centered_it_*time_it_) = Ten-year trajectory of blood pressure according sex centered for subject i in the time t

_6_(Age centered_it_*time_it_) = Ten-year trajectory of blood pressure according age centered for subject i in the time t

_7_(Bmi_it_*time_it_) = Ten-year trajectory of blood pressure according bmi centered for subject i in the time t

_8_(Education_it_*time_it_) = Ten-year trajectory of blood pressure according education level for subject i in the time t

_9_(Calories_it_*time_it_) = Ten-year trajectory of blood pressure according calorie intake for subject i in the time t

_n_(Covariates) = physical activity, smoking status and alcohol intake.

u_it_ = error term
