# Supplementary material for: Soft drink and non-caloric soft drink intake and their association with blood pressure: the Health Workers Cohort Study
Source: Nutr J. 2022 Jun 7;21:37. doi: 10.1186/s12937-022-00792-y (PMC9171938; doi:10.1186/s12937-022-00792-y)
Supplement: Supplementary file 2 — Additional file 2: Supplementary Table 1. Association of soft drink and non-caloric soft drink intake with blood pressure (mm Hg) in participants without type-2 diabetes at baseline (n=1,241)1. Supplementary Table 2. Association of soft drink and non-caloric soft drink intake with blood pressure (mm Hg) in participants without obesity at baseline (n=1,071)1. [file 12937_2022_792_MOESM2_ESM.docx]

| **Supplementary Table 1.** Association of soft drink and non-caloric soft drink intake with blood pressure (mm Hg) in participants without type-2 diabetes at baseline (n=1,241)^1^ | | | | | | |
| --- | --- | --- | --- | --- | --- | --- |
|  | **Soft drink intake** | | | **Non-caloric soft drink intake** | | |
| **Variable** | **Coefficient** | **CI 95%** | **P-value** | **Coefficient** | **CI 95%** | **P-value** |
| **Systolic blood pressure** |  |  |  |  |  |  |
| Soft drinks | 0.16 | -1.28, 1.60 | 0.83 | 0.25 | -2.49, 3.00 | 0.86 |
| Time | 10.38 | 6.53, 14.22 | <0.001 | 11.45 | 7.75, 15.15 | <0.001 |
| Soft drinks*Time | 1.85 | -0.04 3.74 | 0.06 | -0.23 | -2.53, 2.08 | 0.85 |
| **Diastolic blood pressure** |  |  |  |  |  |  |
| Soft drinks | 0.24 | -0.76, 1.25 | 0.63 | 0.20 | -1.74, 2.14 | 0.84 |
| Time | 5.40 | 2.71, 8.08 | <0.001 | 6.14 | 3.53, 8.76 | <0.001 |
| Soft drinks*Time | 1.81 | 0.48, 3.13 | <0.01 | -0.11 | -1.74, 1.52 | 0.90 |
| ^1^Models were adjusted for age centered to mean, sex centered, body mass index centered to mean, physical activity, smoking status, alcohol intake, education, energy intake. | | | | | | |

| **Supplementary Table 2.** Association of soft drink and non-caloric soft drink intake with blood pressure (mm Hg) in participants without obesity at baseline (n=1,071)^1^ | | | | | | |
| --- | --- | --- | --- | --- | --- | --- |
|  | **Soft drink intake** | | | **Non-caloric soft drink intake** | | |
| **Variable** | **Coefficient** | **CI 95%** | **P-value** | **Coefficient** | **CI 95%** | **P-value** |
| **Systolic blood pressure** |  |  |  |  |  |  |
| Soft drinks | 0.35 | -1.19, 1.90 | 0.46 | -0.70 | -3.52, 2.12 | 0.63 |
| Time | 10.81 | 6.72, 14.90 | <0.001 | 11.86 | 7.91, 15.80 | <0.001 |
| Soft drinks*Time | 2.27 | 0.18, 4.36 | 0.03 | 1.55 | -1.38, 4.48 | 0.30 |
| **Diastolic blood pressure** |  |  |  |  |  |  |
| Soft drinks | 0.01 | -1.04, 1.07 | 0.98 | -0.66 | -2.61, 1.29 | 0.51 |
| Time | 4.90 | 2.11, 7.70 | <0.01 | 5.80 | 3.07, 8.53 | <0.001 |
| Soft drinks*Time | 2.51 | 1.08, 3.94 | 0.001 | 1.57 | -0.46, 3.59 | 0.13 |
| ^1^Models were adjusted for age centered to mean, sex centered, body mass index centered to mean, physical activity, smoking status, alcohol intake, education, energy intake. | | | | | | |
